# A simple method to estimate the number of doses to include in a bank of vaccines. The case of Lumpy Skin Disease in France

**DOI:** 10.1371/journal.pone.0210317

**Published:** 2019-01-25

**Authors:** Jordi Casal, Claude Saegerman, Stéphane Bertagnoli, Gilles Meyer, Jean Pierre Ganière, Philippe Caufour, Kris De Clercq, Philippe Jacquiet, Claire Hautefeuille, Florence Etore, Sebastián Napp

**Affiliations:** 1 Departament de Sanitat i Anatomia Animals. Universitat Autònoma de Barcelona, Bellaterra, Barcelona, Spain; 2 IRTA, Centre de Recerca en Sanitat Animal (CReSA, IRTA-UAB), Bellaterra, Barcelona, Spain; 3 Expert Committee in Animal Health and Welfare from ANSES, Maisons-Alfort, France; 4 Fundamental and Applied Research for Animal and Health (FARAH) Center, University of Liège, Liège, Belgium; 5 IHAP, Université de Toulouse, INRA, ENVT, Toulouse, France; 6 ONIRIS, Nantes, France; 7 UMR Cirad-Inra ASTRE, Department BIOS, CIRAD, Montpellier, France; 8 Unit Vesicular and Exotic Diseases, CODA-CERVA, Ukkel, Belgium; 9 Agence nationale de sécurité sanitaire de l'alimentation, de l'environnement et du travail (ANSES), Maisons-Alfort, France; Erasmus MC, NETHERLANDS

## Abstract

A simple method to estimate the size of the vaccine bank needed to control an epidemic of an exotic infectious disease in case of introduction into a country is presented. The method was applied to the case of a Lumpy Skin disease (LSD) epidemic in France. The size of the stock of vaccines needed was calculated based on a series of simple equations that use some trigonometric functions and take into account the spread of the disease, the time required to obtain good vaccination coverage and the cattle density in the affected region. Assuming a 7-weeks period to vaccinate all the animals and a spread of the disease of 7.3 km/week, the vaccination of 740 716 cattle would be enough to control an epidemic of LSD in France in 90% of the simulations (608 196 cattle would cover 75% of the simulations). The results of this simple method were then validated using a dynamic simulation model, which served as reference for the calculation of the vaccine stock required. The differences between both models in different scenarios, related with the time needed to vaccinate the animals, ranged from 7% to 10.5% more vaccines using the simple method to cover 90% of the simulations, and from 9.0% to 13.8% for 75% of the simulations. The model is easy to use and may be adapted for the control of different diseases in different countries, just by using some simple formulas and few input data.

## Introduction

A big concern for the national veterinary authorities in all countries is the preparedness for the introduction of an exotic disease. A good and rapid response to a new disease can represent an important reduction in the cost of the disease due to the lower number of animals that would become affected and to the earlier eradication of the disease. This last aspect is of paramount importance to reduce the trade restrictions, especially for countries that export large number of animals.

The preparedness includes different aspects such as surveillance, establishment of preventive measures (especially in the borders), the elaboration of contingency plans, and the storage of all the material that may be needed in case of a sanitary crisis. One of the items to be stocked for a rapid control of some exotic diseases are the vaccines. Initially, vaccine banks were composed of bulk reserves of vaccines ready to use, but it has evolved to storing concentrated inactivated antigens over liquid nitrogen that allow having vaccines ready to use in a short period of time [1] Several countries and consortia of countries have banks of different vaccines, concentrated antigens or similar products. Forman and Garland [2] cited 16 vaccine banks for foot and mouth disease (FMD), 12 of which were national and the other four operated on an international basis. The EU keeps also a vaccine bank for the control of classical swine fever (CSF). The Word Organization for Animal Health (OIE) has a broad experience in the management of vaccine banks for different diseases. By October 2017, they have three active vaccine banks (for Rabies in Africa and Asia, FMD in South-East Asia and Peste des Petits Ruminants (PPR) in West Africa) [3]

Ring vaccination is a useful control measure in case of an outbreak of an exotic disease. It has been applied in different countries to fight against FMD [4] or CSF. Also for Lumpy Skin disease (LSD) in Iraq, where all herds located within a 10 km radius around the outbreaks were vaccinated [5]. In some cases, as in the equine influenza outbreak in Australia during the 2007, the zone has been modified to account for local conditions [6].

The objective of this paper is to describe a simple method to estimate the size that a vaccine bank should have to manage an emergency vaccination campaign in case of introduction of a given exotic disease in a particular country. We applied the method to evaluate the number of vaccines that would need to be stocked for the control of a LSD epidemic in France. Finally, we validated this simple method by calculating the number of vaccines needed in that case (LSD epidemic in France) using a more complex methodology.

## Material and methods

### Lumpy skin disease

Lumpy skin disease (LSD) is caused by a virus of the *Poxviridae* family, genus *Capripoxvirus* [7]. The virus affects cattle, buffalos and zebus. LSD is categorized as a notifiable disease by the World Organization for Animal Health (OIE) because of its significant economic impact. The most typical clinical sign is the presence of nodules in the skin. The nodules either regress, or progress to necrosis, ulcers and finally scars. These lesions can appear also in other tissues in the respiratory, digestive and genital tracts, as well as in the lymph nodes [8]. The most important method of transmission is by blood-feeding vectors that would mechanically transmit the virus. The spread of the disease within areas is usually in clusters, with sporadic jumps over long distances.

Nowadays, there is a consensus between animal health authorities and researchers that a combination of vaccination and movement restrictions with or without removing clinical affected animals is the best option to control and eradicate LSD [9].

The disease remained endemic in sub-Saharan Africa for decades, but at the end of the last century, it spread through Egypt (1988), the Middle East (1989) and in the recent years it spread again in Middle East (2012), Turkey (2013), Cyprus (2014) and different Balkan countries, Caucasian countries and the Russian Federation (2015–16).

### Scenario

We simulated the number of vaccines needed in case LSD was introduced in France and caused an epidemic. Data on the number of cattle herds and the number of individual cows by department, as well as the surface of those departments were obtained from the annual agricultural statistics from 2015 and from the “Institut National de l’Information Géographique et forestière” from France.

### Model

To determine the size of the stock of vaccines needed to control an epidemic of an exotic disease, the following factors were taken into account:

1- The speed of spread of the disease through the affected area (i.e. distance travelled by the disease per week). The value used for the calculations in the example of LSD in France, was the diffusion rate at which the LSD spread from western Turkey to the Balkan countries between May 2015 and August 2016. This spread was estimated as 7.3 km / week [10].
2- The time between the introduction of the disease and the accomplishment of an effective vaccination of the bovine population, which implies the development of a protective immune response in an adequate proportion of the population (i.e. good vaccination coverage). For the calculation of this time, three periods need to be considered:
  ▪ the time elapsed between the introduction of the disease and its detection, and the start of the vaccination campaign (2–4 weeks),
  ▪ the time needed to vaccinate the entire target population (1–2 weeks), and
  ▪ the time needed for animals to achieve an effective protection after vaccination (1–2 weeks).
For LSD in France, we estimated that the time for effective vaccination may vary between 4 weeks (best case-scenario) and 8 weeks (worst case-scenario).
3- The cattle density in the zone where the first outbreak of the exotic disease was detected. Densities were calculated by departments (the second level administrative division in France). The risk of introduction of LSD in the country is mainly linked to the introduction of infected live cattle from an affected area. As the number of animals coming from affected areas imported into the country is very low, and both the numbers of animals imported and the areas affected vary across time, there is no point in using that data for the simulations. Therefore, we assumed that LSD could be introduced in any part of France, and that the risk of the first outbreak occurring in a given department was directly proportional to the population size in each department. That allowed us to evaluate the whole range of possible scenarios after the introduction of LSD into France.

Another assumption was that all cattle present in the area would be vaccinated. The calculations can be refined according to the ages of the animals to be immunized and to the actual vaccination capacities in the field.

The product of the three values: speed of diffusion, delay between infection and effective vaccination of all the population and density of cattle gives the size of the stock of vaccines to be stored:

- Estimation of the density of cattle in each department (Dep.) and each region (Reg.):

$$Density\;Dep.=\frac{Number\;of\;cattle\;in\;the\;Dep.}{Surface\;of\;the\;Dep.}$$ (1)

$$Density\;Reg.=\frac{Number\;of\;cattle\;in\;the\;Reg.}{Surface\;of\;the\;Reg.}$$ (2)

The first outbreak appears in a department that was chosen using random numbers weighed by the cattle census. To simplify the calculations, the department was considered as a square with a side of:

$$Side=\sqrt{Surface}$$ (3)

Another random number defines the location inside the department. If the radius of the vaccination zone exceeds the limits of the department, vaccination will apply also to the corresponding area in neighbouring departments within the same region. In order to simplify the calculations, it was assumed that the vaccination zone never exceeds the limits of the region affecting another region, the sea or a neighbouring country.

LocalizationFirstoutbreak=Randomnumber×Side (4)

- Estimation of the number of animals that need to be vaccinated.

If the whole vaccination area is inside the department (i.e. the vaccination radius is shorter than the distance between the random number and the limit of the department), the cattle density of this given department is considered.

$$Number\;of\;cattle=Dep.density\;\times \pi \;\times vaccination\;radius^{2}$$ (5)

But, if the vaccination area exceeds the department, the density of the region is also considered:

$$Number\;of\;cattle\;outside\;the\;department\;\left(but\;inside\;the\;region\right)=Region\;Density\;\;\times \left(\varphi -\;\sin(\varphi \right))\times vaccination\;radius{\;}^{2}/2$$ (6)

$$Number\;of\;cattle\;inside\;the\;department=Dep.Density\;\times \left(\pi \times vaccination\;radius^{2}-\left(\varphi -\;\sin(\varphi \right)\right)\times vaccination\;radius^{2}/2)$$ (7)

Where φ is the angle (in radians) formed by the two lines connecting the position of the first outbreak and the two intersections of the circle of the vaccination zone and the side of the department. And it is calculated:

$$\varphi =2\;\times ArcCos\left(\frac{Side\;Dep.\;\;\times \;random}{vaccination\;radius}\right)$$ (8)

**Fig 1:**
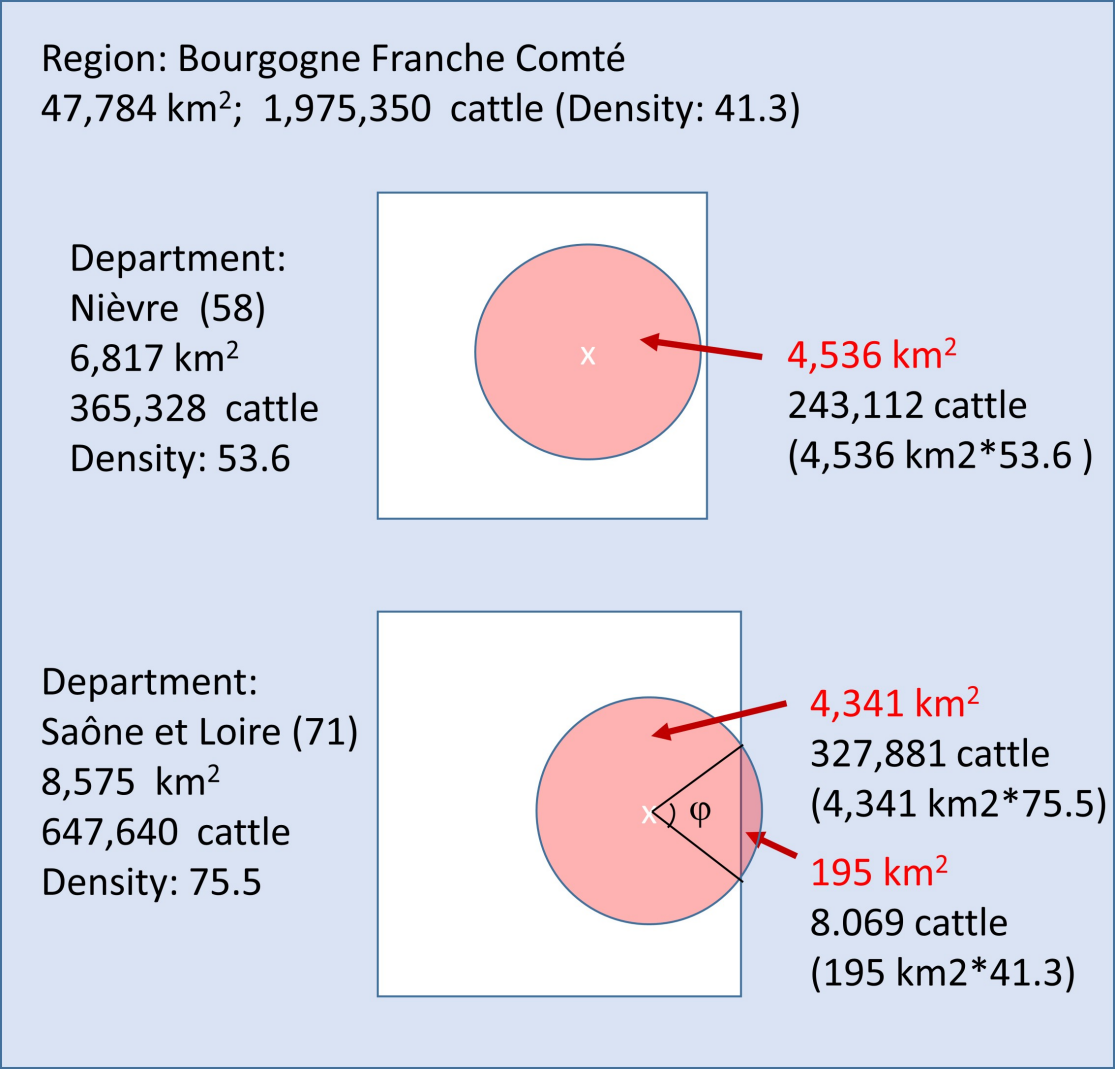
Graphic representation of the calculations to obtain the number of cattle to be vaccinate: In the top, the outbreak appears in Saône-et-Loire department and part of the circle goes beyond the department, affecting other departments of the region. In the bottom the vaccination area is completely inside the Nièvre department.

Fig 1 shows two examples to illustrate both situations for the departments of Saône-et-Loire (Dep. 71) and la Nièvre (58) at the Bourgogne Franche Comté region.

The model was built in an Excel Spreadsheet, using a macro to simulate 20000 iterations (S1 File).

### Validation of the results

For the validation of the results of the model, we also built a dynamic model based on the real geographical characteristics of France, which we used as a reference (S1 and S2 Texts and S2 File). In order to so, first all French cattle herds within each department were randomly allocated within that department. That allowed us to create a map of France with the approximated location of each of the herds in the country (i.e. accounting for the heterogeneity in cattle density across departments). All herds within each department were assumed to have the same size, obtained by dividing the number of cows in that Department by the number of herds.

In a second step, LSD spread was simulated. In order to do so, one farm in France was randomly selected as the index case of the disease. Then, the disease was assumed to spread at a given speed (7.3 Km per week) [10], i.e. the radius of the circular area affected by the disease increased at that speed. Therefore, the number of herds and animals included within that area (i.e. potentially affected by the disease) was simulated for each week (from week 1 up to week 20). The process of selecting a random herd and simulating disease spread was repeated for a thousand times to account for the fact that the disease may start in any herd of France, and that the consequences (number of herds and animals affected) will vary depending on the density of herds and animals in that area.

All simulations were carried out using R software [11]. Packages sp [12], rgeos [13], dplyr [14] and tidyr [15] were used for the development of the dynamic model.

## Results

**Fig 2:**
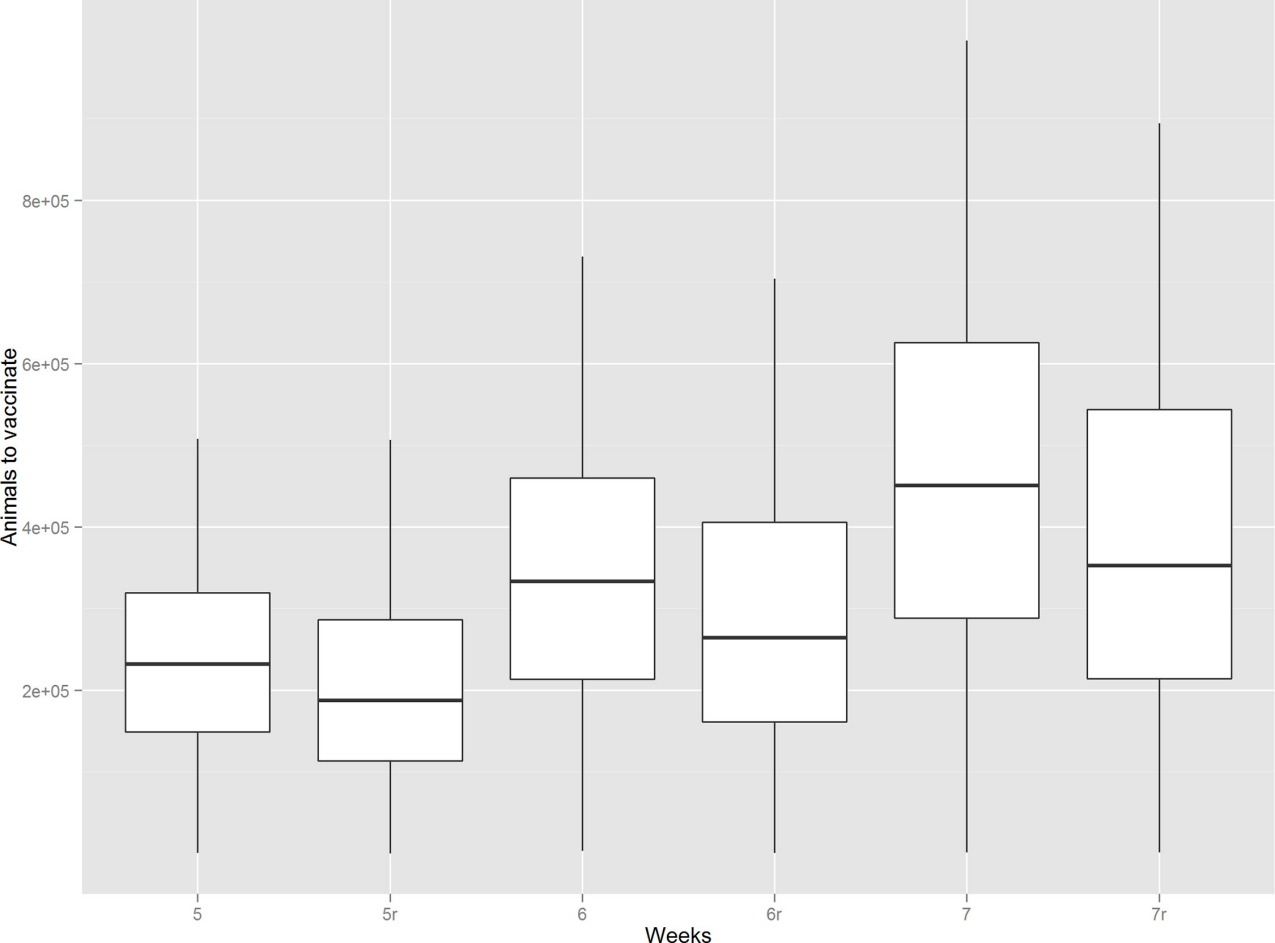
Boxplot with the number of cattle to be vaccinated according to the time (from 5 to 7 weeks) between the detection of the first case and the whole vaccination of the zone. 5r, 6r and 7r represent the results obtained with the refined method (numbers without letter indicate the values of the simple method).

Table 1 shows, for both the simple and the dynamic model, the estimated percentiles 50, 75, 90 and 95 of the animals that would be included in the area of vaccination in case of LSD introduction into France, and given a disease-spread period of between 4 and 8 weeks. For a 7 weeks period between the introduction of LSD and the implementation of an effective vaccination, the vaccination area would have a radius of 51.1 km around the index case (8,203 km^2^). For this period, 90% of the simulations using the simple model included 740 716 cattle or less. If we consider the 75% of the simulations, the number of cattle to vaccinate would be 608 196. Using the dynamic model, the number of animals estimated to need vaccination would be 678 328 and 544 324 respectively (9.2 and 11.7% higher for the simple model than for the reference model respectively). For the different periods of time considered between onset of the disease and the end of the vaccination, the difference ranges from 7% to 10.5% for the values representing the 90% of the simulations and 9.0%-13.8% for 75% of the simulations. For the values close to the median (as well as for the very extreme values, i.e. percentile 95) the simple method tends to overestimate the number of cattle that would need vaccination. The distribution of the number of cattle to be vaccinated according to the two methods is depicted in Fig 2.

**Table 1:** Number of cattle to be vaccinated according the time between the first outbreak and the last animal of the area become immunized. Percentages (50% to 95%) indicate the corresponding percentiles for that week. In bold the number of animals estimated using the simplified method, in italics using the refined method, and the difference between them.

Weeks	Radius	Method	50%	75%	90%	95%
4	29.2	**Simple**	**147903**	**200238**	**244905**	**301616,5**
		*Refined*	*122907*	*186456*	*232150*,*4*	*267157*,*7*
		Difference	20.3%	7.4%	5.5%	12.9%
5	36.5	**Simple**	**229640**	**312011**	**382094**	**464799**
		*Refined*	*188015*	*286339*	*357068*	*405460*
		Difference	22.1%	9.0%	7.0%	14.6%
6	43.8	**Simple**	**327074**	**447711**	**546728**	**660538**
		*Refined*	*264875*	*405724*	*505105*	*564229*
		Difference	23.5%	10.3%	8.2%	17.1%
7	51.1	**Simple**	**442474**	**608196**	**740716**	**894534**
		*Refined*	*352950*	*544324*	*678328*	*744464*
		Difference	25.4%	11.7%	9.2%	20.2%
8	58.4	**Simple**	**577119**	**795938**	**965141**	**1149848**
		*Refined*	*453062*	*699144*	*873654*	*955775*
		Difference	27.4%	13.8%	10.5%	20.3%

Table 2 shows the departments with higher number of cattle in the vaccination area. If the first outbreak appears in Manche and Mayenne, about one million of doses would be needed to guarantee 90% of the simulations for a 7 weeks period, for the reference method the number would be significantly lower (near to 800 000 doses), for the other departments the differences are smaller.

**Table 2:** Departments with higher number of doses needed to vaccinate the whole population in 90% of the simulations considering periods of 6 and 7 weeks between infection and vaccination, and according the two methods of calculation (simple method and the reference method).

	Period of 6 weeks			Period of 7 weeks		
Department	Simple model	Dynamic model	difference (%)	Simple model	Dynamic model	difference (%)
Manche	751,984	583,263	29	1,029,076	770,710	34
Mayenne	735,707	624,342	18	1,005,020	827,239	21
Ille-et-Vilaine	577,384	585,450	-1	788,052	779,620	1
Vendée	549,055	452,639	21	746,617	584,645	28
Cantal	502,568	461,072	9	690,894	606,018	14
Creuse	470,231	513,101	-8	644,676	711,497	-9
Côtes d'Armor	467,306	436,708	7	635,977	578,289	10
Maine-et-Loire	463,469	478,781	-3	631,177	622,878	1
Loire Atlantique	463,343	434,377	7	631,694	587,808	7
Orne	461,668	501,317	-8	628,509	686,677	-8

## Discussion

The preparedness for the introduction of exotic diseases in a country is a big challenge as, in the case of an epidemic of an exotic disease, the economic consequences may be devastating. Vaccination is the most effective control measure for several diseases. In those cases, the availability of the right number of vaccine doses in a short period of time is of paramount importance. The best way to cope with a contingency of that kind is the creation of a vaccine bank with a sufficient number of vaccine doses. The storage of more doses than needed represents a waste of resources, while the storage of less doses than needed may risk the control of the disease. Therefore, an accurate estimation of the number of vaccine doses that may be required in case of the introduction of a given disease is essential.

In the case of LSD introduction into a free country, the best option for its control, is the vaccination of the cattle population and the prevention of animal movements in affected areas. The objective of a vaccination campaign is the immunization of a high enough proportion of the susceptible population. For this reason, we assumed that the goal of an emergency vaccination should be to cover the 100% of the cattle population. In areas previously vaccinated, young calves should not be vaccinated because they can have colostral antibodies. However, in previously free, non-vaccinated areas, all animals, including calves, need to be vaccinated, as did the Greek authorities in 2015 [16].

In case of a LSD epidemic in France, a delay of 4–8 weeks between the declaration of the first case and the achievement of the whole vaccination, would suppose that the number of cattle in the vaccination area would be between 245,000 and 965,000 heads for 90% of the simulations. We have used the speed of spread calculated by Mercier et al [10]. They considered two patterns of dispersion, a local spread associated with the vectors, and a transmission to herds located at medium distances possibly related to cattle movements. However, we have not considered the transmission to large distances, in this case we assume that another ring vaccination procedure should be started, probably with a smaller radius because the risk period before detection would be also shorter. In case of secondary outbreaks located outside of the radius, the number of vaccines would be higher, but we assume that after the detection of the first case, the authorities will start the process to buy more vaccines, and these cases could be vaccinated with doses bought after de onset of the disease.

Besides giving an idea of the size of the stock of vaccines needed, the model may also help to get an idea of the amount of personnel and material resources that would need to be allocated to control the epidemic, and that is key for preparedness for the epidemic.

On the other hand, LSD is a vector borne disease, which means that it is highly seasonal with little transmission during the winter period; the values presented here are adapted to the worst-case scenario, i.e. between May and August with the maximum vector activity [10].

Ring vaccination has been previously applied to fight against FMD, CSF and other diseases. Related to LSD, a ring vaccination with a 10 km radius around the outbreaks was applied in Iraq[5]. Even though a circular protection zone may be a starting point when control policies are planned, local geographical factors have to be considered. Geographical features as rivers, mountains or roads may be responsible for an irregular distribution of cases. Rivas et al. [17] applied a model including roads that required 20% less areas compared to the application of a single radius.

The advantage of the model we present is its simplicity and the facility to adapt it to different conditions (e.g. different diseases or countries). It only requires data on the spread of the disease, the time needed to vaccinate and the census. For 90% of the simulations, the number of animals included in a radius between 36 and 58 km around the first affected farm is 7% - 10.5% higher using the simplest method than with the more accurate method used as reference. Besides, this model is easy to build just by using some simple formulas based on trigonometric functions and with few data. Models that are more complex will imply the use of software that may be complicated and will require the knowledge of programming.

## Supporting information

S1 File Spreadsheet file with the simple model. This is an Excel file containing a macro.
(XLSM)

S2 File Census data for the dynamic model. It is a self-made dataset (a csv file) with the structure needed for the census data to run the model.
(CSV)

S1 Text R script with the dynamic model code. (TXT)

S2 Text Instructions to run the R code. (TXT)
